# Gender Dysphoria and Sexual Euphoria: A Bayesian Perspective on the Influence of Gender-Affirming Hormone Therapy on Sexual Arousal

**DOI:** 10.1007/s10508-023-02778-1

**Published:** 2024-01-12

**Authors:** Manfred Klöbl, Murray Bruce Reed, Patricia Handschuh, Ulrike Kaufmann, Melisande Elisabeth Konadu, Vera Ritter, Benjamin Spurny-Dworak, Georg S. Kranz, Rupert Lanzenberger, Marie Spies

**Affiliations:** 1 Department of Psychiatry and Psychotherapy, Medical University of Vienna, Waehringer Guertel 18-20, 1090 Vienna, Austria; 2 Comprehensive Center for Clinical Neurosciences and Mental Health, Medical University of Vienna, Vienna, Austria; 3 Department of Obstetrics and Gynecology, Medical University of Vienna, Vienna, Austria; 4 Department of Rehabilitation Sciences, The Hong Kong Polytechnic University, Hong Kong, China

**Keywords:** Sexual arousal, Transgender, Gender-affirming hormone therapy, Ventral striatum, Functional magnetic resonance imaging, Bayesian analysis

## Abstract

Self-reported sexual orientation of transgender individuals occasionally changes over transition. Using functional magnetic resonance imaging, we tested the hypothesis that neural and behavioral patterns of sexual arousal in transgender individuals would shift from the assigned to the experienced gender (e.g., trans women’s responses becoming more dissimilar to those of cis men and more similar to those of cis women). To this aim, trans women (*N* = 12) and trans men (*N* = 20) as well as cisgender women (*N* = 24) and cisgender men (*N* = 14) rated visual stimuli showing male–female, female–female or male–male intercourse for sexual arousal before and after four months of gender-affirming hormone therapy. A Bayesian framework allowed us to incorporate previous behavioral findings. The hypothesized changes could indeed be observed in the behavioral responses with the strongest results for trans men and female–female scenes. Activation of the ventral striatum supported our hypothesis only for female–female scenes in trans women. The respective application or depletion of androgens in trans men and trans women might partly explain this observation. The prominent role of female–female stimuli might be based on the differential responses they elicit in cis women and men or, in theory, the controversial concept of autogynephilia. We show that correlates of sexual arousal in transgender individuals might change in the direction of the experienced gender. Future investigations should elucidate the mechanistic role of sex hormones and the cause of the differential neural and behavioral findings.

The study was registered at ClinicalTrials.gov (NCT02715232), March 22, 2016.

**Supplementary Information:**

The online version contains supplementary material available at 10.1007/s10508-023-02778-1.

## Introduction

Self-reported sexual orientation and/or attraction has been found to change for transgender persons during their transition process (Auer et al., 2014; Katz-Wise et al., 2016; Meier et al., 2013). In a study by Auer et al. (2014), 33% of trans women (TW) and 22% of trans men (TM) reported a change in sexual orientation. Of those reporting changes, 39% of the TW and 60% of the TM reported that the changes occurred prior to sex reassignment surgery. Self-reported changes were more likely in individuals previously attracted to the opposite biological sex (i.e., gynephilic TW and androphilic TM) compared with those previously attracted to the same biological sex (i.e., androphilic TW and gynephilic TM). A study by Meier et al. (2013) found that originally gynephilic TM were especially likely to report a change in sexual attraction, suggesting an opposite pattern. In that study, overall 40% of all TM who had begun transition reported a change in sexual orientation. Despite being contradictory, these results point toward an interaction between gender and pre-transition sexual preferences in mediating the temporal dynamics of self-reported sexual attraction/orientation over transition. These factors must thus both be taken into account when assessing changes to sexual arousal over transition.

The use of myriad self-report and physiological measures is limiting comparability between studies. In addition, inter-individual variance in genital arousal patterns is high, particularly in case of bisexuality (Slettevold et al., 2019). Also, patterns of sexual attraction in self-report or physiological measures might not always be interchangeable with sexual orientation, resulting in cases where, e.g., self-reported sexual orientation changes but patterns of sexual arousal do not (Lawrence et al., 2005). Similarly, behavioral and peripheral (genital) measures do not necessarily carry over to brain activity patterns associated with sexual arousal. For example, differences between cis men (CM) and cis women (CW) in subjective arousal (Murnen & Stockton, 1997) were not detected when assessing brain activity during sexual arousal with functional magnetic resonance imaging (fMRI) (Mitricheva et al., 2019). This apparent contradiction between different measures of sexual arousal might be related to a desynchrony in genital and subjective response to erotic stimulation, which might even be stronger in CW than in CM (Meston & Stanton, 2019; Sierra et al., 2019). Thus, comparative investigations of sexual arousal should consider different measures to capture the full spectrum of responses. Moreover, besides genital measures, for which an ascertainment bias needs to be taken into account (Chivers et al., 2004), neural responses might provide an alternative way to quantify sexual arousal (Ponseti et al., 2006).

Regarding the brain regions involved in sexual arousal, only the hypothalamus and the ventral striatum (VS) showed activation specifically corresponding to sexual and not general emotional arousal (Walter et al., 2008). The VS has been employed extensively for investigating brain correlates of sexual arousal across genders (Safron et al., 2007, 2017, 2020; Sylva et al., 2013), whereas hypothalamus activity is specifically related to male sexuality (Brunetti et al., 2008; Karama et al., 2002). Furthermore, the hypothalamus is functionally heterogeneous and sexually dimorphic (Makris et al., 2013), making it a suboptimal fMRI target (Ogawa et al., 2020; Osada et al., 2017). It was shown that gender and sexual orientation not only influence the magnitude of the behavioral and VS response but also its specificity for the stimulation material (Safron et al., 2017, 2020). However, longitudinal studies with transgender individuals investigating the influence of transition in general, or gender-affirming hormone therapy (GHT) in particular, on the changes in brain function related to sexual arousal are missing.

While studying transgender individuals receiving GHT offers the possibility to jointly investigate the roles of assigned gender, experienced gender, sexual orientation, sex hormones and social aspects of transition on sexual arousal (Kranz et al., 2020), insufficient emphasis has been placed on the confounding influence of sex hormones on brain function when their effect was not of primary interest (for reviews, see Heany et al. (2016); Slettevold et al. (2019)). Beyond the effect of sex hormones on neural activity, their effect on sexual behavior is well known (Schober & Pfaff, 2007). For instance, low-dose androgen therapy can improve sexual function in post-menopausal women (Vegunta et al., 2020) and hypogonadal men (Corona et al., 2016), whereas decreased testosterone or increased estrogen levels are associated with erectile dysfunction (Schulster et al., 2016). Moreover, testosterone use was also associated with changes in sexual attraction in TM but not independent of their preferences before beginning transition (Meier et al., 2013).

Here we assess changes to brain measures of sexual arousal related to transition using fMRI, focusing on the VS, based on previous reports of its specific role in this function (Safron et al., 2007, 2017, 2020; Sylva et al., 2013; Walter et al., 2008). We hypothesized that the patterns of behavioral responses and VS activation to erotic stimuli shift from assigned to experienced gender over the course of GHT, in analogy to patterns detected using other MRI based outcome parameters, such as white and gray matter structure (Flores et al., 2022; Kilpatrick et al., 2019; Kranz et al., 2017, 2018). To this aim, we assessed the subjective arousal ratings and VS activation in a sample of TW and TM before and after four months of GHT as well as CW and CM control groups, all undergoing an fMRI sexual arousal task. We then tested whether the rating and VS activation patterns of the TW and TM became more similar to the experienced and more dissimilar to the assigned gender.

## Method

### Participants

Transgender individuals seeking GHT were recruited from the Unit for Gender Identity Disorder, Department of Gynecology and Obstetrics at the General Hospital in Vienna. Cisgender control subjects were recruited via social media, designated message boards at the Medical University of Vienna and from a Neuroimaging Labs database. CM and CW were age-matched ± 3 years to TW and TM, respectively. Inclusion criteria comprised: a diagnosis of gender dysphoria (TM, TW only) according to the *Diagnostic and Statistical Manual of Mental Disorders*, version 5 (DSM-5: 302.85); general health based on medical history, physical examination, electrocardiogram, laboratory screening and structural clinical interview (SCID) for DSM-IV Axis-I. Participants were excluded in case of major neurological or internal illnesses, pregnancy, severe Axis-I comorbidities (TM, TW) or any Axis-I disorder (CM, CW), steroid hormone treatment within six months prior to inclusion (including hormonal contraceptives, with the exception of low dose progesterone in TM for cessation of menstruation prior to transition), treatment with psychotropic agents three months prior to inclusion, clinically relevant abnormal laboratory values, MRI contraindications, current substance abuse (excluding nicotine), current or past substance-related disorders and insufficient compliance.

### Procedure

The study followed a controlled longitudinal observational design. All participants underwent two MRI sessions with an interval of (median ± interquartile range) 138 ± 35 days. After the first MRI, hormone treatment following an individualized regimen according to protocols of the Department of Obstetrics and Gynecology at the Medical University of Vienna was initiated. MRI sessions included structural, diffusion-weighted, task, resting-state, and spectroscopy scans. Only the task data are presented here. Blood for determining testosterone, progesterone, and estradiol plasma levels was drawn on the day of the MRI assessments. The Klein Sexual Orientation Grid was administered 14 ± 24 days before the first and 14 ± 23 days after the second MRI appointment.

### Measures

In the MRI scanner, participants were visually presented a sequence of blocks with five explicit images of male–female, female–female or male–male intercourse. Sports scenes with two women (sports–female) or two men (sports–male) constituted the control for general arousal. Each image was shown for 4 s. Participants were instructed to indicate whether they are “strongly turned on,” “turned on,” “turned off,” or “strongly turned off” by the erotic stimuli using fingers of their right hand and an MRI-compatible four-button response device. For the sports scenes, participants were instructed to indicate how much they liked the image since rating them for arousal led to confusion in pilot runs. Four smiley faces with strong positive to strong negative expressions were depicted below the stimuli and circled in red after pressing the respective button. The 20-s blocks were flanked with baseline periods of the same length showing a fixation cross in the center of the stimulus frame. The task contained four blocks of each sexual arousal and two of each control condition in random order. The stimuli from Safron et al. (2007, 2017), Sylva et al. (2013) and normative scores were kindly provided by the authors. Since the material did not contain male–female scenes, we added these from a database of pornographic movie stills. This was done in order to prevent generalized aversive reactions to homosexual stimuli, which might be expected for the CW group (Sylva et al., 2013). An internal validation ensured comparable style and explicitness to the existing stimuli.

MRI scanning was conducted on a Siemens Prisma 3 T machine: TE/TR = 30/2050 ms, parallel acquisition with GRAPPA 2, 210.0 × 210.0 × 121.8 mm field of view with 100 × 100 pixel in-plane resolution and 35 axial slices of 2.8 mm (25% gap), resulting in 2.1 × 2.1 × 2.8 mm voxel size, flip angle 90°, 2275 Hz/pixel bandwidth, orientation parallel to the anterior–posterior commissure line.

### Data Analysis

#### fMRI Preprocessing and Modeling

Data preprocessing was conducted using PESTICA (Beall & Lowe, 2007), SPM12 (http://www.fil.ion.ucl.ac.uk/spm/; RRID:SCR_007037) and the BrainWavelet Toolbox (Patel et al., 2014) (see supplement for single steps and parameters).

Single subject models contained one regressor per condition (male–female, female–female, male–male, sports–female, sports–male), the Friston-24 model of motion (Friston et al., 1996), and an automatically derived number of combined white matter and cerebrospinal fluid regressors for an adapted CompCor approach (Behzadi et al., 2007; Klöbl et al., 2020). Contrasts for raw erotic (male–female, female–female, male–male) and sports scenes (sports–female, sports–male) as well as category specific activations (|male–female—female–female|, |male–female—male–male|, |male–male—female–female|, i.e., the absolute difference between the *raw* erotic stimuli) were calculated. Even though previous works employed sports scenes to control for general arousal when investigating only a single sex or gender (e.g., Arnow et al., 2002, 2009; Safron et al., 2007, 2017), we refrained from contrasting the erotic to the sports stimuli for three reasons: first, the above-mentioned necessity of different instructions, second, the reactions to watching sports is gender-specific (Apostolou et al., 2014; Gantz & Wenner, 1991), and third, VS activation might vary with the sex of the athletes (Sylva et al., 2013). However, we provide an analysis of the contrasts between erotic and sports stimuli in the supplement.

Median activation was extracted from the VS region in the Automated Anatomical Labeling atlas 3 (AAL3) (Rolls et al., 2020) using the MarsBaR toolbox, version 0.44 (http://marsbar.sourceforge.net/; RRID:SCR_009605). Despite its activation specific to sexual arousal, the hypothalamus was not investigated as target region due to its heterogeneous structure.

#### Statistical Modeling and Inference

To test whether behavioral and neural (VS) response patterns shift from assigned to experienced gender, Bayesian multivariate mixed-effects models (brms_2.15.0 R package (Bürkner, 2017) run in RStudio 1.4.1717 (http://www.rstudio.com/; RRID:SCR_000432)) were used as statistical framework for our analyses for the following reasons: First, we aimed to incorporate previous behavioral results from comparable studies in order to partly compensate for the typically low sample sizes in studies on transgender populations. Second, the posterior probabilities (PPs) provided by Bayesian statistics facilitate more fine-grained interpretations, in accordance with the spectrum perspective on gender. In other words, though Bayesian models do not provide *p*-values for dichotomous decisions on significance, they provide probabilities on the outcomes of tests. The PP quantifies the support for our hypotheses after the model “has seen” the data, with higher percentages indicating stronger support, though no cutoff, such as the 5% typically used for the *p*-value. Third, complex hypotheses like measures of sexual arousal approaching the experienced gender and simultaneously distancing from the assigned gender are not easily evaluable in many other approaches, whereas the brms package allows for non-linear hypothesis testing. Bayesian multilevel models further protect against multiplicity issues (Gelman et al., 2012), which would have arisen for frequentist comparisons of four groups and two time points, allowing for more in-depth inferences. See Flores et al. (2022) for a detailed comparison of frequentist versus Bayesian mixed models and the supplement for a complementary frequentist analysis.

It was first tested whether the responses of all transgender participants over all sexual arousal conditions shifted from the assigned to the experienced gender. This means that the response pattern of TW would become more dissimilar to that of CM and more similar to that of CW. In turn, the response pattern of TM would become more dissimilar to that of CW and more similar to that of CM.

Second, since neither similar behavior between sexes and genders, nor across fMRI stimulus groups (i.e., male–female, female–female, and male–male scenes) can be assumed, sub-hypotheses separating TW and TM as well as the stimuli were further evaluated. We first investigated behavioral and VS category specificities of sexual arousal since this was shown to differ between sexes and orientations (Safron et al., 2017, 2020). Category specificity is calculated as the absolute difference between the responses to varying stimulus types (i.e., $\left| {{\text{male}}{-}{{{\text{female}}}} - {\text{female}}{-}{{{\text{female}}}} } \right|$, $\left| {{\text{male}}{-}{{{\text{female}}}} - {\text{male}}{-}{{{\text{male}}}} } \right|$, $\left| {{\text{male}}{-}{{{\text{male}}}} - {\text{female}}{-}{{{\text{female}}}} } \right|$) and thus denotes how different responses to the individual stimulus types are. Subsequently, since no particularly strong evidence was found for changes in category specificity of sexual arousal, the raw responses, i.e., the responses to all five conditions (i.e., male–female, female–female, male–male, sports–female, sports–male) were considered separately to unveil whether the absence of more specific effects resulted from generally comparable responses, added variance or a general lack of effects. Lastly, since the VS is generally sensitive to rewarding stimulation (which might be given by seeing individuals of a particular sex) and there might be latent driving factors underlying subjective ratings not covered by the experiment, we conducted an arousal-specific analysis, contrasting the erotic to the sports stimuli. The shifts in responses and VS activation in all three cases above were formally assessed using the following inequation:

$$ \left| {{\text{TM}}_{{{\text{post}}}} - {\text{CM}}_{{{\text{post}}}} } \right| + \left| {{\text{TW}}_{{{\text{post}}}} - {\text{CW}}_{{{\text{post}}}} } \right| + \left| {{\text{TM}}_{{{\text{pre}}}} - {\text{CW}}_{{{\text{pre}}}} } \right| + \left| {{\text{TW}}_{{{\text{pre}}}} - {\text{CM}}_{{{\text{pre}}}} } \right| < \left| {{\text{TM}}_{{{\text{pre}}}} - {\text{CM}}_{{{\text{pre}}}} } \right| + \left| {{\text{TW}}_{{{\text{pre}}}} - {\text{CW}}_{{{\text{pre}}}} } \right| + \left| {{\text{TM}}_{{{\text{post}}}} - {\text{CW}}_{{{\text{post}}}} } \right| + \left| {{\text{TW}}_{{{\text{post}}}} - {\text{CM}}_{{{\text{post}}}} } \right| $$

The sexual preference of the participants at each time point was quantified as the mean of the Klein Sexual Orientation Grid items “current sexual attraction,” “current sexual behavior,” and “current sexual phantasies,” which were rated on a 7-point Likert scale from “women only” to “men only.” The score was inverse-coded for CW and TW to reflect the distance to heterosexuality with respect to the experienced gender in all groups, mean-centered per group and time point and used as covariate in all models to account for the effects on orientation-specific sexual arousal. This also controls for potentially mediating effects of the initial sexual preferences of the transgender participants (Auer et al., 2014; Meier et al., 2013). Furthermore, assuming that there is a shift in sexual arousal over the observed transition period while receiving GHT, the temporal dynamics of this effect will vary between individuals. We thus used the above-mentioned items of the Klein Sexual Orientation Grid to account for varying magnitude and rate of the hypothesized effect. Participant age was mean-centered within groups as well to avoid collinearity due to differences between the groups. To correct for the varying GHT regimens, the first principal components of the standardized log-transformed testosterone, progesterone and estradiol levels were included as separate covariates for TM and TW at the second measurement since group-specific effects were likely (Mueller et al., 2020). Missing hormone and Klein Sexual Orientation Grid values were estimated using the Missing Data Imputation toolbox, version 2, in MATLAB (Folch-Fortuny et al., 2016) (see supplement for details). The models further contained fixed group and time point factors, their interactions, and a random intercept per participant with correlations between the conditions. Per protocol, subjects were assessed after approximately 4 months of GHT (median ± interquartile range = 127 ± 14.25 days). We did not adjust for the time of receiving gender-affirming hormone therapy, as this was comparable between the subjects. Priors for behavioral data were calculated based on the sexual orientation score, the results of Gizewski et al. (2009), Ku et al. (2013), Safron et al. (2007), and the normative data from Safron et al. (2007) kindly provided by the authors (see supplement for mathematical details). To examine the influence of the priors on the behavioral results, we additionally conducted a complementary analysis using flat priors (as for the VS activation) presented in the supplement. Pairwise comparisons of the factors gave 95% highest posterior density (HPD) regions, i.e., areas of the posterior distributions containing 95% of the estimated samples. The nonlinear hypothesis tests return posterior probabilities of support by the data.

## Results

The demographics and hormone levels of the study sample are provided in Table 1. Overall, data from 20 TW, 12 TM, 24 CW, and 14 CM was included in this analysis. Partially available data from the first session was included from ten participants. For two participants, only the second session could be used due to a date recording error. Less TW and matched CM could be recruited compared to TM and CW. Due to age matching, the former groups are also older. On average, subjective responses indicate that male–female stimuli were received as turning on over all groups except for TM before undergoing GHT. In contrast, male–male stimuli were received as turning off except for TM after undergoing GHT. The female–female stimuli show a marked difference between the cisgender groups with CW being turned off and CM being turned on. TW and TM reported an increase in subjective sexual arousal for female–female stimuli. At the second assessment, TW had numerically lower testosterone and higher progesterone and estradiol levels, whereas TM had higher testosterone, lower progesterone and lower estradiol levels. The hormone levels of the cisgender groups were more stable with slight variations in progesterone as well as estradiol in CW only. Demographics and covariates grouped by individuals participating only in the first or both MRI sessions are presented in the Supplementary Table S1.

**Table 1:** Demographics, behavioral data, and covariates

	Cis women	Cis men	Trans women	Trans men
N pre	23	13	12	20
N post	21	10	9	20
Age [years]^a,b^	23.29 ± 5.40	27.27 ± 5.56	26.63 ± 3.51	22.30 ± 7.94
Preferences pre^c^	7.00 [6.00, 7.00]	1.00 [1.00, 1.33]	2.00 [1.00, 5.00]	2.00 [1.00, 6.33]
Preferences post^c^	7.00 [5.67, 7.00]	1.00 [1.00, 1.33]	2.67 [1.00, 6.67]	2.33 [1.00, 7.00]
Male–female pre^b,d^	0.94 ± 1.15	1.60 ± 0.43	0.72 ± 1.52	0.04 ± 1.86
Male–female post^b,d^	0.93 ± 1.15	1.67 ± 0.85	0.68 ± 1.60	0.75 ± 2.21
Female–female pre^b,d^	− 0.50 ± .1.49	1.21 ± 1.54	0.70 ± 1.52	− 0.44 ± 1.76
Female–female post^b,d^	− 1.00 ± 1.57	1.23 ± 0.71	1.35 ± 2.45	0.38 ± 1.79
Male–male pre^b,d^	− 1.30 ± 1.39	− 1.95 ± 0.35	− 1.32 ± 1.31	− 1.18 ± 2.61
Male–male post^b,d^	− 1.35 ± 1.48	− 1.92 ± 0.96	− 1.58 ± 0.95	0.22 ± 2.24
Sports–female pre^b,d^	− 1.50 ± 1.45	− 0.40 ± 1.00	− 1.05 ± 1.38	− 0.05 ± 1.52
Sports–female post^b,d^	− 1.80 ± 0.89	− 0.70 ± 2.57	− 1.10 ± 1.98	− 0.65 ± 2.37
Sport–male pre^b,d^	− 1.20 ± 1.30	− 0.90 ± 0.50	− 0.95 ± 0.82	− 0.15 ± 1.17
Sport–male post^b,d^	− 1.33 ± 1.00	− 1.80 ± 2.00	− 1.50 ± 0.70	− 0.53 ± 1.82
Testosterone pre [ng/ml]^b,e^	0.33 ± 0.18	4.80 ± 2.93	5.36 ± 1.57	0.40 ± 0.22
Testosterone post [ng/ml]^b,e^	0.35 ± 0.21	4.99 ± 0.99	0.30 ± 0.20	3.66 ± 2.10
Progesterone pre [ng/ml]^b,e^	0.63 ± 1.24	0.27 ± 0.50	0.26 ± 0.28	2.29 ± 6.10
Progesterone post [ng/ml]^b,e^	0.87 ± 8.81	0.18 ± 0.14	0.19 ± 0.89	0.31 ± 0.17
Estradiol pre [pg/ml]^b,e^	55.00 ± 71.50	23.00 ± 14.0	35.00 ± 17.00	79.84 ± 101.00
Estradiol post [pg/ml]^b,e^	75.00 ± 102.00	21.50 ± 12.25	121.00 ± 466.00	42.39 ± 32.00

^a^At first measurement. ^b^Median ± interquartile ranges (third minus first). ^c^Median [minimum, maximum]; calculated from the Klein Sexual Orientation Grid with 1 = women only and 7 = men only. ^d^Interval [− 2 = strongly turning off, 2 = strongly turning on]; not corrected for sexual orientation here. ^e^Hormone levels for cisgender participants and pre-treatment time points provided for comparison only and not included in the statistical analysis

### Subjective Sexual Arousal Ratings

**Fig. 1:**
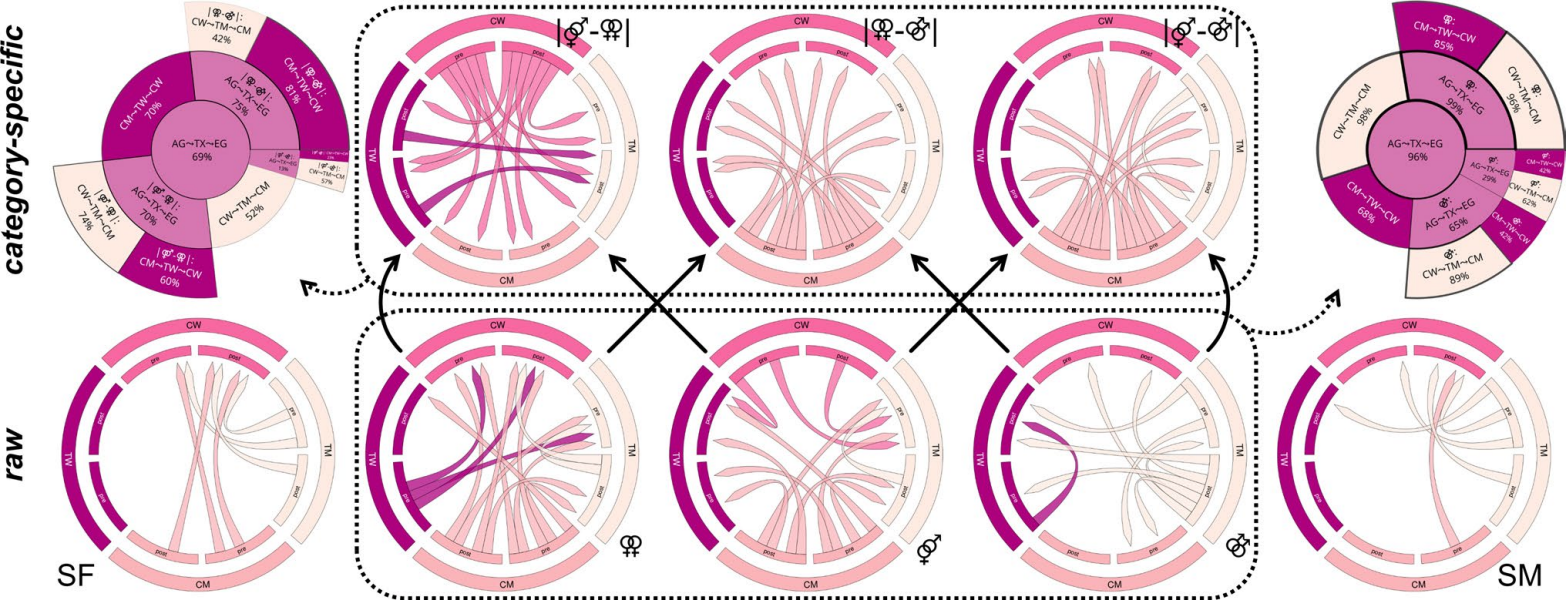
Results of the behavioral analyses. For visualization purposes, the chord diagrams (Gu et al., 2014) only show the differences between groups and time points with a 95% highest posterior density not covering 0 (this is very roughly similar to a corrected significance of *p* < .05). The top row shows the category specific and the bottom row the raw differences. Colored arrow directions in the chord diagrams indicate “greater than” relations (i.e., the arrows represent the “>” relation with the smaller value at the tip and the larger at the shaft). For instance, the top left chord diagram shows that the category specificity of CW for heterosexual versus female–female stimuli is greater than for almost all other groups and time points, whereas, for TW at both time points, it is greater only than that of TM after the observed period of gender-affirming hormone therapy. Black solid arrows between the diagrams show which raw results fed into the calculation of the category specific results. Dotted lines and arrows indicate the results used for hypothesis testing. The hypothesis tests for response patterns shifting from the assigned to the experienced gender are presented on the top left (category specific responses) and right (raw responses) with their respective posterior probabilities. The central circle represents the support for the main hypothesis with the sectors fanning out representing the derived sub-hypotheses. For instance, the support for our hypothesis of a shift from the assigned to the experienced gender including all raw stimuli and groups was 96%, where it was even 99% for female–female stimuli and 98% for TM. For the responses of TM to female–female stimuli, it was 96%. The results for the sports scenes showing female (SF) or male (SM) athletes were not included in the hypothesis tests. AG: assigned gender, TX: transgender, EG: experienced gender, TW: trans women, TM: trans men, CW: cis women, CM: cis men, pre: pre-treatment assessment, post: post-treatment assessment, ⚤: scenes of male–female intercourse, ⚢: scenes of female–female intercourse, ⚣: scenes of male–male intercourse

The behavioral model showed more pronounced category specificity for the comparisons male–female versus male–male and female–female versus male–male stimuli for CM at both time points (visualized for 95%-HPDs not covering zero in Fig. 1). The most category specificity for female–female versus male–female stimuli was found for CW, again at both time points. Category specificity for female–female versus male–female stimuli was stronger in TW than TM. The category specificity for male–female versus male–male stimuli decreased for TM, whereas the raw response increased for all erotic stimulus types.

Category specific behavioral data provided weak to moderate support for our hypothesis of transgender sexual arousal response patterns moving from the assigned to the experienced gender. This was strongest in TW for the category specificity of female–female versus male–male (PP = 81%) and weak in TW and TM for the category specificity of male–female versus male–male stimuli (PP = 23% and PP = 57%, respectively). In summary, after GHT, TW became more similar to CW and/or more dissimilar to CM in their varying responses (i.e., category specificity) to female–female versus male–male stimuli.

Raw behavioral data clearly showed the hypothesized changes. This effect was especially strong for female–female stimuli (PP = 99%) in both TW (PP = 85%) and TM (PP = 96%). TM showed the hypothesized shift in sexual arousal patterns across all erotic stimuli categories (PP = 98%) but least for the male–female stimuli (PP = 62%). An overview of the behavioral results including PPs showing to which degree the data supports our hypothesis is given in Fig. 1. Moderate to strong correlations of random intercepts were found for raw male–female and female–female (*r* = 0.57, 95% credible interval [0.32, 0.76]), male–female and female sports (*r* = 0.36, [0.01, 0.68]), female–female and female sports (*r* = 0.42, [0.06, 0.73]), and female sports and male sports scenes (*r* = 0.73, [0.33, 0.95]; see supplement for full data).

### Ventral Striatum Activation

**Fig. 2:**
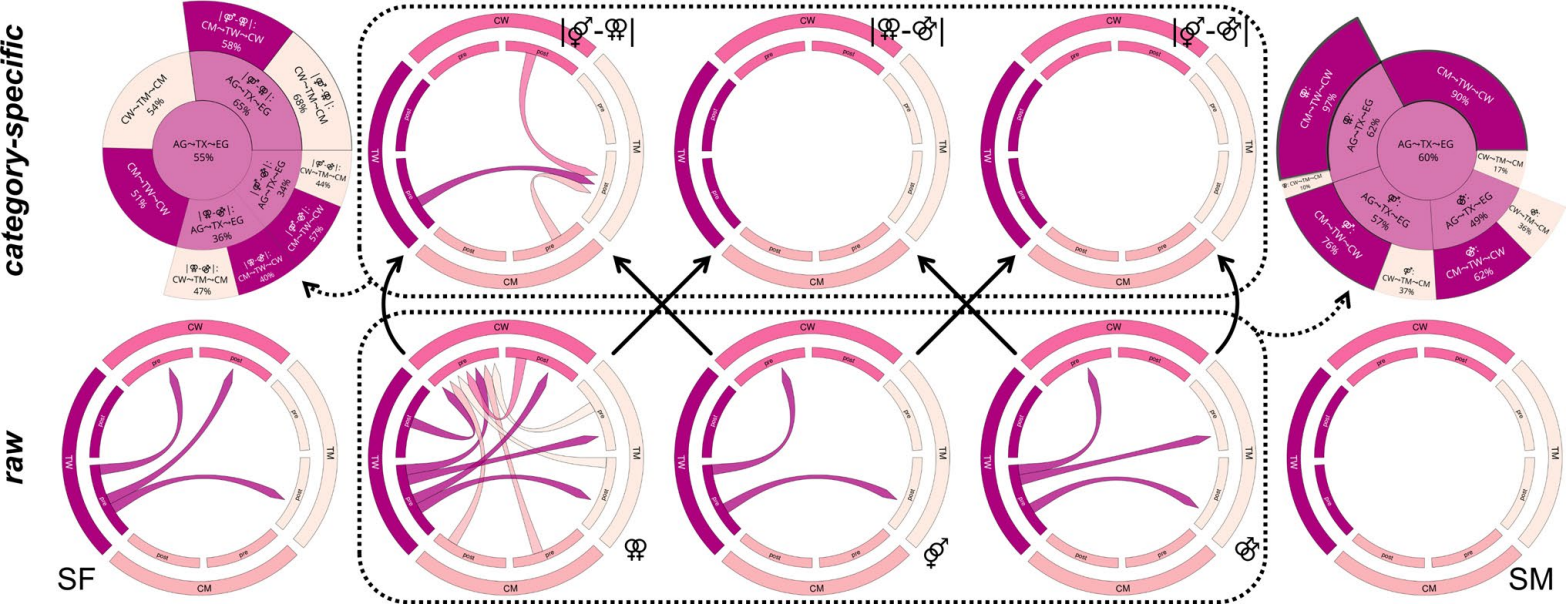
Results of the ventral striatum activation analyses. For visualization purposes, the chord diagrams (Gu et al., 2014) only show the differences between groups and time points with a 95% highest posterior density not covering 0. The top row shows the category specific and the bottom row the raw differences. Colored arrow directions in the chord diagrams indicate “greater than” relations (i.e., the arrows represent the “>” relation with the smaller value at the tip and the larger at the shaft). Black solid arrows between the diagrams show which raw results fed into the calculation of the category specific results. Dotted lines and arrows indicate the results used for hypothesis testing. The hypothesis tests for ventral striatum activation patterns shifting from the assigned to the experienced gender are presented on the top left (category specific activation) and right (raw activation) with their respective posterior probabilities. The central circle represents the support for the main hypothesis with the sectors fanning out representing the derived sub-hypotheses. The results for the sports scenes showing female (SF) or male (SM) athletes were not included in the hypothesis tests. AG: assigned gender, TX: transgender, EG: experienced gender, TW: trans women, TM: trans men, CW: cis women, CM: cis men, pre: pre-treatment assessment, post: post-treatment assessment, ⚤: scenes of male–female intercourse, ⚢: scenes of female–female intercourse, ⚣: scenes of male–male intercourse

After four months of GHT, TM displayed clearly weaker category specificity of VS activation for male–female versus female–female stimuli than the other groups (see 95%-HPDs in Fig. 2). Distinct changes in the raw data according to our hypotheses were found mostly for TW (PP = 90%), especially with female–female stimuli (PP = 97%). An overview of the results for VS activation is given in Fig. 2. Correlations between the categories were weaker with considerably larger credible intervals. Complete details on the models and tests are provided in the supplementary tables.

## Discussion

We investigated whether the behavioral and neural responses to sexual arousal in transgender individuals shift from the assigned to the experienced gender over the course of four months of GHT. Overall strong support for this hypothesis was found in the raw behavioral responses, especially for the TM group and female–female scenes. For VS activation, a comparable effect was observed for female–female stimuli presented to the TW group. The support for category specific changes was markedly weaker as indicated by the lower PPs.

In general, the cisgender groups showed markedly stronger category specificity of their behavioral responses than the transgender groups. We included male–female stimuli to avoid receiving primarily aversive responses from CW (Sylva et al., 2013). Indeed, the most prominent behavioral category specificity among all subject groups and stimulus types was found for male–female versus female–female scenes in CW and male–female or female–female versus male–male scenes in CM at both time points. These findings are in accordance with Safron et al. (2020), insofar as that they show strong male–male versus female–female category specificity for CM. Our results now extend the strong category specificity of CM toward male–female versus male–male stimuli. In contrast, CW exhibit strong category specificity for male–female versus female–female stimuli, where this group signaled subjective arousal only for male–female images.

### Effects in Trans Men

After four months of GHT, the TM group displayed a strong reduction in category specificity of male–female versus male–male stimuli as well as an increase spanning all raw responses to erotic stimuli. Furthermore, VS activation showed particularly less category specificity for male–female versus female–female stimuli after GHT compared to CM and TW at the first and CW at the second time point. The raw increases were absent in the neural data. Altogether, these results provided at most weak support for our hypothesis on category specific responses in TM shifting over GHT, but might point toward generally higher subjective sexual arousal after treatment. This interpretation is in line with previous reports of increased sexual desire and arousal in TM after sex reassignment surgery and GHT (Mueller et al., 2020) and might be explained by the general positive relationship between sexual arousal and testosterone levels (Klein & Gorzalka, 2009).

The lack of further category specific changes in TM might be attributable to a stronger expression of bisexualism among androphilic and ambiphilic/bisexual transitioned TM compared to CM with the same orientations (Bockting et al., 2009). Even though we adjusted our analyses for self-reported orientation using the Klein Sexual Orientation Grid, such traits might exist beyond what could be captured by the questionnaire. While measures of genital arousal displayed traits similar to CW and CM in a mixed pre- and post-surgical TM sample and the male-typical preference for orientation-congruent stimuli was present, more female-typical response to all stimuli was nevertheless observed (Raines et al., 2021). Our TM sample identified as mostly gynephilic, but existing bisexual rather than monosexual traits could partly explain the lack of clear changes in category specific responses to sexual arousal.

Of note, TM also had the least aversive behavioral responses to the sports scenes. Despite the correlations found between erotic and sports stimuli, a latent influence of general emotional arousal is unlikely, since the participants were instructed to rate the sports stimuli on how much they liked the image in general and not for sexual arousal (see section Measures). The observation that TM and CM responded the most positively to the sports scenes with the strongest difference to CW is potentially based on the generally higher appeal of watching sports in men. The evolutionary perspective of sports as a primarily male communication tool (Apostolou et al., 2014) as well as gender-related social norms and roles (Gantz & Wenner, 1991) might provide explanations here. Data from transgender individuals on the experience of watching sports is missing but our results suggest a difference between TW and TW as well as a potential influence of four months of GHT (see chord diagrams in Figs. 1 and 2 and also supplementary tables). Specific research on this topic is, however, necessary to confirm or refute these deliberations.

The raw behavioral responses to the erotic stimuli provide general support for our hypothesis, particularly with the TM pattern becoming more CM-/less CW-typical across erotic stimuli of all orientations. In detail, the increase in arousal for female–female scenes can be explained by the response approaching a CM and for the male–male scenes by distancing from a CW pattern. On the contrary, the VS activation does not show any changes in the hypothesized direction. This stark difference between behavioral and neural measures offers two potential explanations: First, the activation for sexual arousal does not change in the VS for TM after GHT. Second, there is incongruence between subjectively perceived and neurally controlled level of arousal. In an fMRI study with accompanying behavioral data, a small sample of gynephilic-leaning post-operative TM showed a significantly higher level of reported arousal to pictures showing two nude women compared to nude men (Kim et al., 2016a, 2016b). Activation of the hypothalamus, but not the VS was reported. Where the hypothalamus was found, like the VS, to react to sexual rather than general arousal (Walter et al., 2008), this reaction is potentially sex-specific and predominantly found in men (Brunetti et al., 2008; Karama et al., 2002). While the hypothalamus was not investigated here, this might be done in future studies on a voxelwise basis or after refining the target region (Osada et al., 2017), keeping structural sex differences in mind (Makris et al., 2013).

### Effects in Trans Women

The TW group showed stronger behavioral category specificity for male–female versus female–female scenes compared to the TM group at and across both time points. This effect is also found for VS activation but only for pre-treatment TW and post-treatment TM. Pre-treatment, TW’s raw response to female–female stimuli was more positive than that of the CW or the TM group before GHT, establishing a certain similarity to the CM group. Contrary to TM, before undergoing GHT, TW showed increased raw VS activation for all conditions except the male sports scenes. This general level of VS activation and the resulting similarity across stimuli also explains the lack of neural category specificity in TW. Despite weaker changes on the behavioral level, the neural data indicates some shift from the assigned toward the experienced gender in TW, which was strongest for female–female scenes.

In a study on post-operative TW, premenopausal and menopausal CW, similar activation patterns to nude pictures were found in the former two but not in the last group (Kim & Jeong, 2014). These results should be interpreted with caution due to the age difference between groups (despite correction) and its highly exploratory nature. However, they point toward similarities in TW after transitioning and CW, as we hypothesized in our data set, as well as hormonal influences on sexual arousal. A large fMRI study comparing transgender individuals living in their experienced gender to cisgender controls reported significantly lower activation for erotic stimuli in the right superior temporal gyrus for TW as compared to CM (Mueller et al., 2020). The investigation was based on the “neurophenomenological model of sexual arousal” proposed by Stoléru et al. (2012). According to this model, the superior temporal gyrus belongs to the “inhibition and devaluation” component, the VS to the “motivational” component (like the hypothalamus). Interestingly, among all groups, the highest interhemispheric task connectivity for the superior temporal gyrus was also found in TW. Given the relationship between VS activation and whole-brain functional connectivity (Mori et al., 2019), seed-based analyses might shed further light on its particular importance for sexual arousal in TW.

The commonalities in behavioral and neural responses (i.e., both being strongest for female–female stimuli) could, in theory, be related to the controversial concept of autogynephilia in TW (Blanchard, 1989). While the self-reported orientation of our TW sample is gynephilic on average before and after GHT (Auer et al., 2014), the perceived sexual arousal by watching the intercourse of other women might have experienced a potentially hormone-induced actualization. Laube et al. (2020) reported a statistical trend that the desire for intercourse with a partner was particularly strong in gynephilic TW (as was our sample on average) before undergoing sex reassignment surgery and, to a lesser extent, before GHT. If such an increased sexual desire were mirrored in stronger VS activation, this would provide an alternative perspective on our findings to autogynephilia: The comparably weaker VS activation in TW after undergoing GHT matches the gradient in sexual desire indicated by Laube et al. (2020). In turn, the weaker VS activation as well as diminished sexual desire are in line with the argumentation of Blanchard (1991) that the effects of estrogen treatment and surgical castration reduce the sex drive in TW. Another possible explanation for the VS activation shift in TW strongest for female–female stimuli would be the group’s generally higher activation levels being reduced after GHT and this coinciding with the strongest difference in activation between CM and CW for female–female stimuli. In total, this could create the impression that the VS activation of the TW group shifts from a CM to a CW pattern only for female–female scenes.

Autogynephilia could, in theory, explain the higher VS activation in TW group to the female sports stimuli before undergoing GHT, which is in stark contrast to the more positive subjective ratings of the TM group, explainable by stronger interest in sports. The recently formulated “brain gender dissonance model” (Altinay & Anand, 2020) hypothesizes a close relationship between VS activation and cognitive dissonance in transgender individuals for stimuli inducing gender dysphoria. This might have happened in TW particularly before undergoing GHT when viewing the female sports scenes since these lacked a stronger stimulation such as sexual content. The authors of the model admit that the relation between VS and cognitive dissonance has not yet been investigated in transgender individuals. However, previous studies found correlations between attitude changes after decisions inducing cognitive dissonance (e.g., having to decide between two similarly liked options) and VS (Jarcho et al., 2010) and caudate nucleus activation (sometimes seen as part of the VS; Sharot et al. (2009)).

Whether these findings are comparable to cognitive dissonance induced by gender dysphoria and what this means for stimuli of coinciding hedonic content and cognitive dissonance needs to be answered by future research. Further points needing consideration are the more negative experience of sexuality in TW (Gil-Llario et al., 2021) and the suppression of female sexuality per se (Muggleton et al., 2019). Both could introduce biases in the neural and behavioral correlates of sexual arousal and might have contributed to the lack of behavioral effects.

### Limitations

Less TW than TM could be recruited, which is in line with the reported incidence of gender-confirming surgery (Nolan et al., 2019), and a considerable amount of TW dropped out of the study. Even though this seems to be a general trend, there are societal and age-related influences on the ratio of gender dysphoria in biological males and females. The higher estimated prevalence of gender dysphoria in assigned male gender might be driven by the more complex developmental sex differentiation processes on a hormonal but also psychosocial level (Zucker, 2017). However, psychosocial factors might also lead to a distortion of the true prevalence ratio. Since we also found support for our hypothesis in TW, this likely did not lead to an underpowered sample. Still, simplifications were necessary when correcting for the potential influence of the varying treatment regimens. Since this study on transgender individuals rather than sexual preferences, mixed orientations are found especially in the TW and TM groups. Also, some cisgender participants might be described as heterosexual-leaning rather than strictly heterosexual. Correcting for the influence of varying sexual orientation leads to results for average responses, which are gynephilic-leaning in both transgender groups. Lastly, it is not possible to conclude whether the effects found solely stem from the influence of the hormones on brain and behavior or result, from an increased self-perception of the experienced gender, or from a wide range of potential social and personal influences during transition. In addition, due to methodological restrictions, we assumed categorical, not fluid gender, i.e. the extent to which individuals may experience themselves as belonging to a certain gender would affect experienced sexual orientation.

### Conclusion

In line with our hypothesis, we propose that raw behavioral and neural measures of sexual arousal in transgender individuals might approach the pattern of the experienced gender during GHT. These effects were considerably stronger for raw responses (especially for female–female stimuli) than for the category specificity of stimuli (i.e., how specific the responses were for one orientation of the stimuli compared to another). Potential explanations for the more pronounced evidence within the raw responses might be, that the arousal to different stimulus categories changed in the same direction or, that the participants’ diverse sexual orientations together with the contrasting of their responses introduced additional variance. Where behavioral analyses showed generally strong support for our hypothesis (slightly stronger in TM), for VS activation, this was only the case in TW, with the most prominent changes found for female–female scenes. While we cannot offer a definite explanation for this observation, the general difference between male–female CM and CW responses could provide increased sensitivity to changes for this category. In TW, the controversial concept of autogynephilia could also offer a perspective. GHT may also play a role in facilitating some of our results. Stronger reported arousal across all erotic stimuli orientations within the TM group might be attributable to the effect of testosterone, which is known to increase libido. In a similar fashion, the diminished VS activation in the TW group might be caused by the application of anti-androgens. Here, we abstained from analyzing the hypothalamus due to its functional heterogeneity and sex-specific organization. However, future studies particularly focusing on this region might shed light on its role in transgender individuals, since the hypothalamus constitutes a promising target given its role in male sexuality. Furthermore, in-depth analyses of the role of the sexual orientation in the transitioning process would shed more light on changes in perceived sexuality.

### Supplementary Information

Below is the link to the electronic supplementary material.

Supplementary file1 (DOCX 12722 KB)

Supplementary file2 (XLSX 28 KB)

Supplementary file3 (XLSX 22 KB)

Supplementary file4 (XLSX 28 KB)

Supplementary file5 (XLSX 22 KB)

Supplementary file6 (XLSX 23 KB)

Supplementary file7 (XLSX 28 KB)

Supplementary file8 (XLSX 40 KB)

Supplementary file9 (XLSX 29 KB)

Supplementary file10 (XLSX 40 KB)

Supplementary file11 (XLSX 23 KB)

## Data Availability

Due to data protection, preprocessed fMRI and behavioral data are available only upon reasonable request to the corresponding author.

## Code Availability

In-house code is available upon reasonable request to the corresponding author.
