# Supplementary material for: Gender Dysphoria and Sexual Euphoria: A Bayesian Perspective on the Influence of Gender-Affirming Hormone Therapy on Sexual Arousal
Source: Arch Sex Behav. 2024 Jan 12;53(5):1859–71. doi: 10.1007/s10508-023-02778-1 (PMC11106106; doi:10.1007/s10508-023-02778-1)
Supplement: Supplementary file 1 — Supplementary file1 (DOCX 12722 KB) [file 10508_2023_2778_MOESM1_ESM.docx]

Supplementary information for

**Gender Dysphoria and Sexual Euphoria: A Bayesian Perspective on the Influence of Gender-Affirming Hormone Therapy on Sexual Arousal**

# SUPPLEMENTARY METHOD

## Sexual Arousal Task – Privacy and Compliance Measures

Before entering the scanner, participants received written instructions with sample images and performed a short training run to familiarize them with the experiment and degree of explicitness. To further encourage genuine answers, we assured participants that data was going to be anonymized before analysis and the experimenter would not be watching the stimulation screen (the lid of the stimulation laptop was closed).

**FMRI Preprocessing**

For data preprocessing, physiological artifacts were first reduced via PESTICA (Beall & Lowe, 2007). Subsequent steps were performed using SPM12 (http://www.fil.ion.ucl.ac.uk/spm/; RRID:SCR_007037), unless otherwise specified. Slice-timing correction was performed to the temporally middle slice, followed by two-pass realignment of both measurements per subject to the mean image. Images were normalized to the standard space defined by the Montreal Neurological Institute (MNI) and resliced to 2.5 mm isotropic, approximately maintaining the voxel volume (K. Mueller, Lepsien, Möller, & Lohmann, 2017). The BrainWavelet Toolbox (Patel et al., 2014) was used for nonlinear artifact reduction with the “chsearch” parameter set to “harsh” for increased artifact sensitivity and the “threshold” set to “20” due to the application to unsmoothed data with decreased signal-to-noise ratio by GRAPPA acceleration. Images were gray-matter masked using a custom template (Klöbl et al., 2020) and smoothed with a Gaussian kernel of three times the resliced voxel size.

## Arousal-specific Contrasts

Complementary to the analysis of the *category-specific* and *raw* responses and VS activation, we analyzed *arousal-specific* data by contrasting the erotic scenes to the sports scenes showing people of the same genders (eliminating common general arousal). Due to a lack of mixed-gender sports images, we averaged the female and male sports conditions here. This resulted in the following three *arousal-specific* contrasts: male-female – (sports-female + sports-male) / 2, female-female – sports-female, male-male – sports-male.

## Sex Hormones

Luteinizing hormone, follicle-stimulating hormone, progesterone, estradiol, testosterone, sex hormone binding globulin and dehydroepiandrosterone sulfate were determined from venous blood drawn on the days of both MRI session. While only testosterone, progesterone, and estradiol were used in the analyses, the other hormones values were used for imputation purposes.

## Missing Data Imputation

As recommended for the given data structure, trimmed-scores regression (TSR) with the standard settings of the MDI toolbox was used (Folch-Fortuny, Arteaga, & Ferrer, 2015). The number of components with the minimum predicted residual sum of squares (PRESS) was

**Table S1: Demographics and covariates at the first (pre) assessment of subjects with and without the second (post) assessment.**

|  | | **Cis women** | | **Cis men** | | **Trans women** | |
| --- | --- | --- | --- | --- | --- | --- | --- |
|  | | **pre only** | **pre & post** | **pre only** | **pre & post** | **pre only** | **pre & post** |
| N | | 3 | 20 | 4 | 9 | 3 | 9 |
| Age [years] ^a^ | | 21.82 ± 4.54 | 23.36 ± 4.97 | 30.78 ± 3.32 | 24.75 ± 4.68 | 25.44 ± 3.15 | 28.30 ± 2.55 |
| Preferences pre ^b^ | | 6.67 [6.00, 7.00] | 7.00 [6.00, 7.00] | 1.00 [1.00, 1.33] | 1.00 [1.00, 1.33] | 2.00 [1.00, 5.00] | 2.00 [1.00, 5.00] |
| Male-female pre ^a,c^ | response | 1.60 ± 1.12 | 0.92 ± 1.00 | 1.70 ± 0.44 | 1.60 ± 0.33 | -1.42 ± 1.28 | 0.75 ± 0.57 |
|  | act. [a.u.] | -0.05 ± 0.06 | -0.07 ± 0.05 | -0.04 ± 0.05 | -0.05 ± 0.11 | -0.00 ± 0.08 | -0.04 ± 0.10 |
| Female-female pre ^a,c^ | response | -0.35 ± 0.42 | -0.57 ± 1.65 | 0.76 ± 1.17 | 1.21 ± 0.65 | 0.25 ± 0.65 | 1.00 ± 1.65 |
|  | act. [a.u.] | -0.04 ± 0.04 | -0.13 ± 0.05 | -0.05 ± 0.13 | -0.05 ± 0.10 | -0.09 ± 0.07 | -0.01 ± 0.12 |
| Male-male pre ^a,c^ | response | -1.85 ± 0.32 | -1.28 ± 1.53 | -1.80 ± 0.75 | -1.95 ± 0.35 | -1.90 ± 1.23 | -1.25 ± 0.97 |
|  | act. [a.u.] | -0.10 ± 0.13 | -0.08 ± 0.08 | -0.08 ± 0.03 | -0.03 ± 0.06 | -0.07 ± 0.06 | -0.03 ± 0.06 |
| Sports-female pre ^a,c^ | response | -0.60 ± 1.20 | -1.50 ± 1.08 | -0.55 ± 0.63 | -0.40 ± 1.10 | -1.10 ± 1.25 | -1.00 ± 1.10 |
|  | act. [a.u.] | -0.07 ± 0.09 | -0.09 ± 0.18 | -0.02 ± 0.13 | -0.12 ± 0.11 | -0.00 ± 0.09 | -0.04 ± 0.10 |
| Sport-male pre ^a,c^ | response | -0.50 ± 0.67 | -1.35 ± 1.19 | -1.10 ± 0.25 | -0.50 ± 1.20 | -0.30 ± 0.97 | -1.00 ± 0.42 |
|  | act. [a.u.] | -0.02 ± 0.06 | -0.07 ± 0.17 | -0.13 ± 0.09 | -0.07 ± 0.08 | -0.05 ± 0.06 | -0.05 ± 0.11 |
| Testosterone pre [ng/ml] ^a,d^ | | 0.23 ± 0.07 | 0.39 ± 0.19 | 5.64 ± 2.98 | 4.80 ± 2.64 | 4.24 ± 1.43 | 5.69 ± 1.77 |
| Progesterone pre [ng/ml] ^a,d^ | | 0.21 ± 0.21 | 0.81 ± 1.86 | 0.58 ± 0.39 | 0.23 ± 0.24 | 0.32 ± 0.12 | 0.20 ± 0.36 |
| Estradiol pre [pg/ml] ^a,d^ | | 49.00 ± 7.00 | 67.00 ± 82.75 | 32.92 ± 3.86 | 21.62 ± 10.00 | 32.00 ± 10.50 | 39.00 ± 17.00 |

^a^ median ± interquartile ranges (third minus first). ^b^ median [minimum, maximum]; calculated from the Klein Sexual Orientation Grid with 1 = women only and 7 = men only. ^c^ response interval [-2 = strongly turning off, 2 = strongly turning on]; not corrected for sexual orientation here; ventral striatum activation given in arbitrary units (act. [a.u.]). ^d^ hormone levels for cisgender participants and pre-treatment time points provided for comparison only and not included in the statistical analysis.

retained. Since the hormone data was strongly right skewed and to avoid unreasonable negative estimates, imputation of missing values (CW: 8.33%, CM: 20%, TW: 23.50%, TM: 17.22%) was performed on log-transformed values. Back-transformed values were thresholded at 1.5 times the interquartile range from the first and third quartile of the original data to remove strong outliers. Due to distinct sex- and treatment-specific patterns with dependencies between first and second assessment, the time points were treated as different variables and imputation was performed for each group separately. Missing values in the Klein Sexual Orientation Grid (CW: 21.22%, CM: 20.18%, TW: 16.74%, TM: 14.01%) were treated similarly but rescaled and logit-transformed for imputation. To avoid infinite values, the interval [0, 1] was compressed to [1/(2N), 1-1/(2N)], where N is the number of elements in the complete dataset. Refer to Table S1 for an overview of the demographic data, dependent variables and covariates grouped by subjects with only the first and both measurements.

## Specifications of the Bayesian Multilevel Model

The outcome of the VS *response specificity* model was approximated using gamma distributions since the more appropriate folded normal or t-distributions were computationally not feasible and consistently produced divergent transitions in the Markov chains during distribution sampling. Normal distributions were used for the *raw* activation model. The behavioral responses were originally coded {-2, -1, 1, 2} but rescaled to [0, 1] for statistical analysis. Since the absolute value of any difference on this scale is again on this scale, all behavioral data was modeled using zero-one-inflated beta distributions. Each model was run with 10000 iterations (half of them for the warm-up phase) and 4 Markov chains. Convergence was checked visually and via the RHAT statistic. The “adapt_delta” parameter was set to “0.99” to eliminate divergent transitions. In Wilkinson notation, the different multivariate regression models were defined as follows:

$$\begin{matrix} ⚤,⚢,⚣,SW,SM \\ \left| ⚤-⚢ \right|,\left| ⚤-⚣ \right|,\left| ⚢-⚣ \right| \\ ⚤-\frac{SW+SM}{2}, ⚢-SW, ⚣-SM \end{matrix}\sim0+G*M+Age0+tfPca+tmPca+sex\_present0+(1 | p | S)$$

with ⚤: scenes of male-female intercourse, ⚢: scenes of female-female intercourse, ⚣: scenes of male-male intercourse, $SW$: sports stimuli showing women, $SM$: sports stimuli showing men.

For interpretability in R, the following naming conventions were used: $SW$: sports stimuli showing women, $SM$: sports stimuli showing men, $G$: group (i.e., {CW, CM, TW, TM}), $M$: measurement (i.e., {1 = pre-treatment, 2 = post-treatment}), $Age0$: age mean-centered per group, $twPca$: first principal component of standardized log-transformed post-treatment hormone levels for the TW group, $tmPca$: first principal component of standardized log-transformed post-treatment hormone levels for the TM group, $sex\_present0$: present sexual orientation score calculated from the Klein Sexual Orientation Grid and mean-centered per group. The single $0$ indicates that no intercept should be used but all levels of the factors are individually coded for easier formulation of the hypotheses to test. The tailing term $(1 | p | S)$ specifies one random intercept per subject $S$ with correlations $p$ between the outcomes ($p$ used for identifying the correlations has no further meaning).

## Prior Calculation

Where possible, mean and standard deviation for the different subgroups and conditions were taken from Gizewski et al. (2009); Ku et al. (2013); Safron et al. (2007) (read from graphs if no exact values were found) as well as the normative data provided by Safron et al. (2007). The values are presented in Table S1 rescaled to the interval [0, 1], which is the range of the beta distribution used for modeling the responses. Since the participants in Gizewski et al. (2009); Ku et al. (2013), in contrast to those in Safron et al. (2007), only had to indicate positive arousal, the lowest score was treated as 0.5 there. Based on the sexual preference score derived from the Klein Sexual Orientation Grid and the gender assigned at birth, the expected responses for each participant and condition in the current study were calculated. For preferences lying between homo- or hetero- and bisexuality, the responses were interpolated using the modified Akima approach in MATLAB (Akima, 1970). The average of all participants per group was used as prior mean. To avoid producing overly narrow priors, the maximum of the standard deviations for the single participants was used. If the sexual preference score fell between orientations, the maximum of the two neighboring standard deviations was also used. Of note, orientation- and gender-specific mean and standard deviation values were taken from the publications mentioned above and only interpolated to the orientation reflected by the Klein Sexual Orientation Grid items “current sexual attraction,” “current sexual behavior,” and “current sexual phantasies”. This was necessary due to mixed orientations/preferences in our groups. The subjects’ responses had no influence on the priors. The priors for the difference scores (i.e., *category-specific* and *arousal-specific* models) were calculated as differences between the distributions (i.e., difference of means, sum of variances). Transformations from the range of the zero-one-inflated beta distribution of the outcome variables ([0, 1]) to the a normal distribution range ([-∞, ∞]) of the linear predictors was conducted according to (Jørgensen & Pedersen, 1997) using the logit function. For a random variable $X$of a given distribution and a corresponding random variable $Y$ in the target distribution, with $\mathbb{E}$ being the expectancy, $\mathbb{V}$ the variance and $\mathbb{S}$ the standard deviation operator, a function $g$ linking the distributions of $X$ and $Y$, and $x$ being a manifestation of a random variable (in our case a scalar value), the following approximations can be derived using a first-order Taylor expansion:

$$\mathbb{E}\left( Y \right)\approx g\left( \mathbb{E}\left( X \right) \right)$$

$$\mathbb{V}\left( Y \right)\approx{g'\left( \mathbb{E}\left( X \right) \right)}^{2}\mathbb{V}\left( X \right)$$

$$\mathbb{S}\left( Y \right)\approx g'\left( \mathbb{E}\left( X \right) \right)\mathbb{S}\left( X \right)$$

For the transformation from normal to logit scale for the (zero-one-inflated) beta distribution, $g$ is defined as follows:

$$g\left( x \right)=\ln\left( \frac{x}{1-x} \right)$$

$$g'\left( x \right)=\frac{1}{x\left( 1-x \right)}$$

## Complementary frequentist models

The generalized liner mixed models used as basis for testing our hypothesis were reevaluated using the frequentist R package glmmTMB (1.1.8). To allow for estimation of the subjective responses via beta distributions defined over the open interval (0, 1), the values were rescaled to [1/N, 1-1/N] with N being the number of data points in the analysis. For raw VS activation, a Gaussian distribution was used and a gamma distribution for VS category specificity. Of note, the glmmTMB package reports estimates on a different scale than brms used for the main analyses. Thus, without scale transforms, only the following results are directly comparable:

- The 95% credible intervals of the population-level effects not covering 0 can be seen as very roughly equivalent to p-values < 0.05.
- For the estimated marginal means (i.e., pairwise comparisons of the groups at each time point) the upper and lower highest posterior density interval not covering 0 is again very roughly comparable to p-values < 0.05.

When comparing the models, the shrinkage property of the Bayesian estimates as well as the asymmetry of the highest posterior densities need consideration (in contrast, frequentist confidence and Bayesian credible intervals are usually symmetric). Furthermore, while shrinkage does provide protection against false positive results, it is not equivalent to the Tukey correction used for the pairwise comparisons. Unfortunately, frequentist frameworks cannot easily reproduce the nonlinear hypothesis tests providing the PPs quantifying the support for our hypotheses. The results of the frequentist models are provided in the supplementary Excel tables together with the flat prior models.

# SUPPLEMENTARY RESULTS

The complete model and test statistics are provided in the supplementary Excel tables. Beyond the naming conventions given in the section Specifications of the Bayesian Multilevel Model, the following abbreviations were further used: XH: heterosexual stimuli, XL: lesbian stimuli, XG: gay stimuli, GG: assigned gender, EG: experienced gender, TX: transgender.

## *Arousal-specific* Compared to *Raw* Results

Compared to the *raw* model, the parameter estimates for the groups (CW, CM, TW, TM) per time point (pre-treatment, post-treatment) showed absolute (i.e., regardless of the direction) changes ranging from 4.07% to 306.08% with a median of 98.35% for the subjective ratings. For the VS activation, the absolute changes ranged from 96.08% to 667.83% with a median of 185.26%. The larger changes for the VS activation likely result from the originally lower parameter estimates. See the supplementary Excel files for the detailed numbers.

### Subjective Sexual Arousal Ratings

For the female-female – sports-female contrast, the responses of the TM group before undergoing GHT were markedly lower than those of the other groups at both time points. The TM group also showed lower responses than the CW and CM groups for the male-female – average sports contrast. Regarding the hypotheses testing a shift in sexual arousal patterns, weak support remained for the TW group and the contrasts involving female-female stimuli dropped to a comparable level for TW and TM (all with PPs around 70%). A marked increase was seen for the male-female stimuli in TW, which almost doubled to 83% after subtracting the average response to all sports stimuli.

### Ventral Striatum Activation

The VS activation did not show any particularly strong differences between the groups and time points or any support for our hypothesis when contrasting the erotic to the sports stimuli.

## Behavioral Analysis with Flat Priors

To examine the influence of the priors derived from previous publications (Gizewski et al., 2009; Ku et al., 2013; Safron et al., 2007) on the behavioral results, we repeated all analyses with flat (i.e., uninformed) priors. Adding the priors had considerable influences on the parameter estimates for the groups (CW, CM, TW, TM) per time point (pre-treatment, post-treatment). The median absolute changes (and ranges) relative to the flat model for all these parameter estimates and just those with priors added were 5.04% [0.68%, 126.25%] / 20.67% [0.92%, 44.27%], respectively for the *raw* model, 2.03% [0.00%, 688.04%] / 5.99% [1.09%, 79.62%] for the *category-specific* model, and 0.78% [0.00%, 207.88%] / 38.24% [17.69%, 77.42%] for the *arousal-specific* model. Interestingly, the CM group showed higher contrasts in the arousal-specific model. However, the influences of the single estimates on the hypothesis tests might have cancelled out resulting in negligible differences for the hypothesis PPs as shown in Figure S1 compared to Figure 1.

**Table S2: Mean and standard deviation of the responses for the different stimulus conditions per group.**

| **Condition** | **Group** | **Orientation** | **Mean** | **Std** | **Reference/comment** |
| --- | --- | --- | --- | --- | --- |
| male-female | CW | hetero | 0.78 ^a^ | 0.09 | Gizewski, et al. (2009) |
| male-female | CW | bi | 0.73 ^a^ | 0.12 | Ku, et al. (2013) (mixed sex) |
| male-female | CW | homo | 0.68 ^a^ | 0.12 | extrapolated from hetero and bi |
| male-female | CM | hetero | 0.80 ^a^ | 0.10 | Gizewski, et al. (2009) |
| male-female | CM | bi | 0.73 ^a^ | 0.17 | Ku, et al. (2013) (mixed sex) |
| male-female | CM | homo | 0.66 ^a^ | 0.17 | extrapolated from hetero and bi |
| male-female | TM | hetero | 0.72 ^a^ | 0.17 | Ku, et al. (2013) (before treatment, due to similarity to Gizewski, et al. (2009) mixed group used for FtM, all orientations) |
| male-female | TM | bi | 0.72 ^a^ | 0.17 | Ku, et al. (2013) (before treatment, due to similarity to Gizewski, et al. (2009) mixed group used for FtM, all orientations) |
| male-female | TM | homo | 0.72 ^a^ | 0.17 | Ku, et al. (2013) (before treatment, due to similarity to Gizewski, et al. (2009) mixed group used for FtM, all orientations) |
| male-female | TW | hetero | 0.72 ^a^ | 0.11 | Gizewski, et al. (2009) (before treatment; sexual orientation not differentiated in original publication, used for all) |
| male-female | TW | bi | 0.72 ^a^ | 0.11 | Gizewski, et al. (2009) (before treatment; sexual orientation not differentiated in original publication, used for all) |
| male-female | TW | homo | 0.72 ^a^ | 0.11 | Gizewski, et al. (2009) (before treatment; sexual orientation not differentiated in original publication, used for all) |
| female-female | CW | hetero | X ^b^ | X | normative table |
| female-female | CW | bi | X ^b^ | X | normative table |
| female-female | CW | homo | X ^b^ | X | normative table |
| female-female | CM | hetero | 0.85 ^a^ | 0.06 | Safron, et al. (2007), checked with normative table |
| female-female | CM | bi | X ^b^ | X | normative table |
| female-female | CM | homo | 0.14 ^a^ | 0.08 | Safron, et al. (2007), checked with normative table |
| male-male | CW | hetero | X ^b^ | X | normative table |
| male-male | CW | bi | X ^b^ | X | normative table |
| male-male | CW | homo | X ^b^ | X | normative table |
| male-male | CM | hetero | 0.12 ^a^ | 0.06 | Safron, et al. (2007), checked with normative table |
| male-male | CM | bi | X ^b^ | X | normative table |
| male-male | CM | homo | 0.88 ^a^ | 0.06 | Safron, et al. (2007), checked with normative table |
| sports-female | CM | hetero | 0.50 ^a^ | 0.12 | Safron, et al. (2007) |
| sports-female | CM | bi | 0.36 ^a^ | 0.12 | interpolated from hetreo and homo |
| sports-female | CM | homo | 0.21 ^a^ | 0.12 | Safron, et al. (2007) |
| sports-male | CM | hetero | 0.50 ^a^ | 0.08 | Safron, et al. (2007) |
| sports-male | CM | bi | 0.45 ^a^ | 0.17 | interpolated from hetreo and homo |
| sports-male | CM | homo | 0.40 ^a^ | 0.17 | Safron, et al. (2007) |

^a^ mapped from the original scales to [0, 1]. ^b^ only derivable from the not publicly available normative data provided by [masked for peer review].


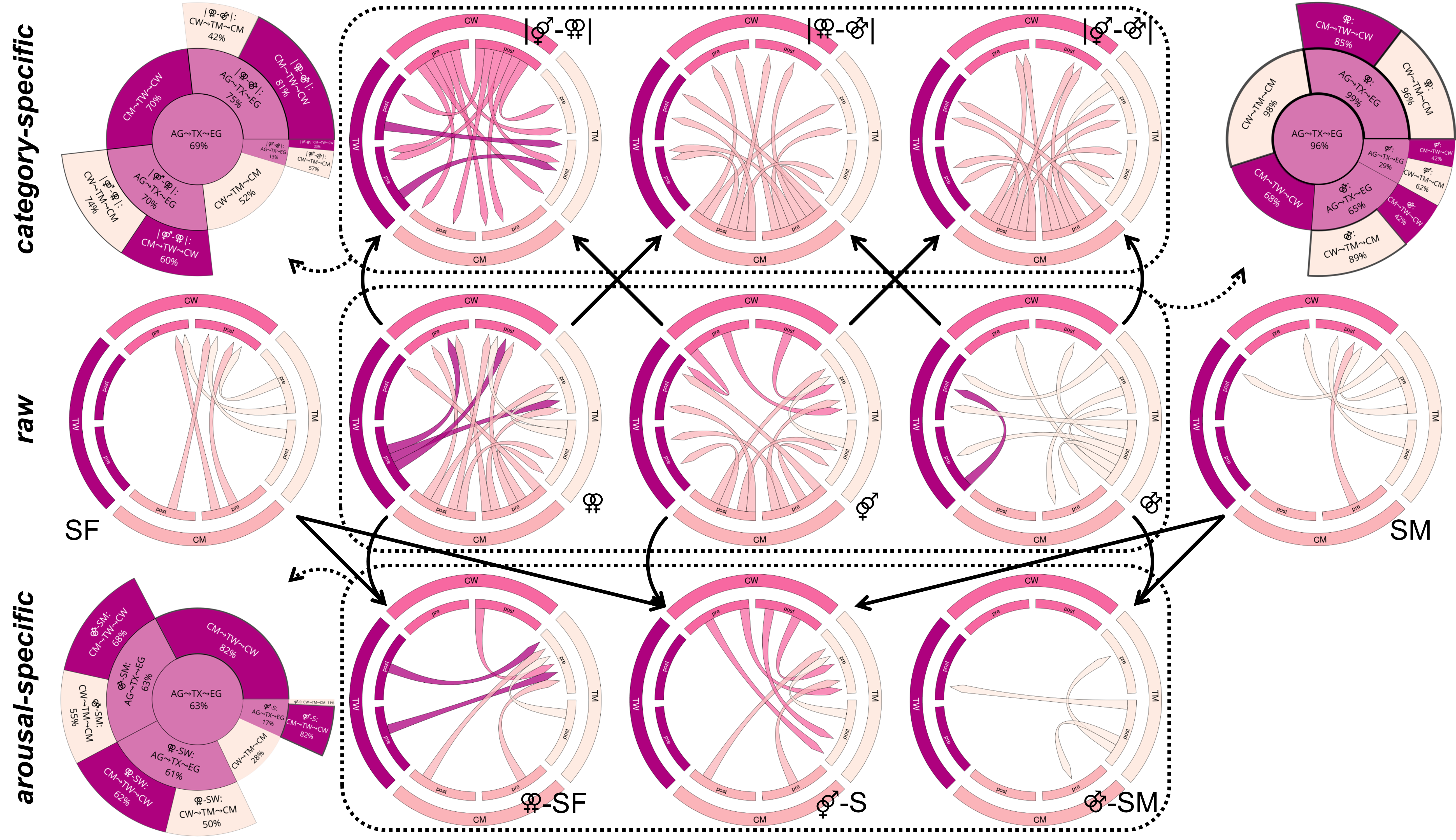


**Figure S1: Results of the behavioral analyses including *arousal-specific* contrasts.** For visualization purposes, the chord diagrams (Gu, Gu, Eils, Schlesner, & Brors, 2014) only show the differences between groups and time points with a 95% highest posterior density not covering 0. The top row shows the *category-specific*, the middle row the *raw*, and the bottom row the *arousal-specific* differences. Colored arrow directions in the chord diagrams indicate “greater than” relations (i.e., the arrows represent the “>” relation with the smaller value at the tip and the larger at the shaft). Black solid arrows between the diagrams show which *raw* results fed into the calculation of the *category-* and *arousal-specific* results. Dotted lines and arrows indicate the results used for hypothesis testing. The hypothesis tests for response patterns shifting from the assigned to the experienced gender are presented on the top left (*category-specific* responses), top right (*raw* responses), and bottom left (*arousal-specific* responses) with their respective posterior probabilities. The central circle represents the support for the main hypothesis with the sectors fanning out representing the derived sub-hypotheses. The results for the sports scenes showing female (SF) or male (SM) athletes were only included in the hypothesis tests of the *arousal-specific* responses. The responses to heterosexual stimuli were contrasted to the average of SW and SM responses (S). AG: assigned gender, TX: transgender, EG: experienced gender, TW: trans women, TM: trans men, CW: cis women, CM: cis men, pre: pre-treatment assessment, post: post-treatment assessment, ⚤: scenes of male-female intercourse, ⚢: scenes of female-female intercourse, ⚣: scenes of male-male intercourse.


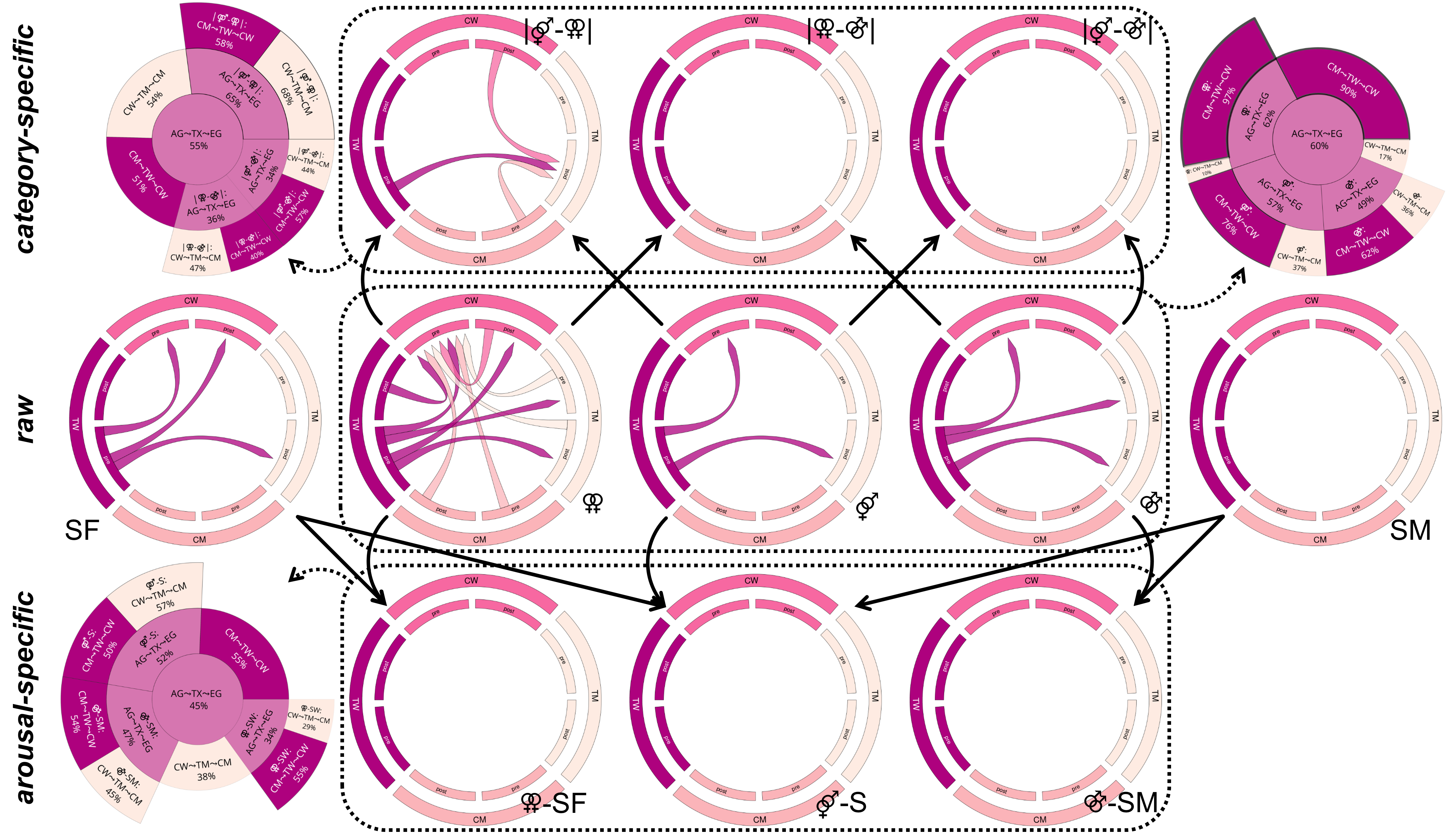


**Figure S2: Results of the ventral striatum activation analyses including *arousal-specific* contrasts.** For visualization purposes, the chord diagrams (Gu et al., 2014) only show the differences between groups and time points with a 95% highest posterior density not covering 0. The top row shows the *category-specific*, the middle row the *raw*, and the bottom row the *arousal-specific* differences. Colored arrow directions in the chord diagrams indicate “greater than” relations (i.e., the arrows represent the “>” relation with the smaller value at the tip and the larger at the shaft). Black solid arrows between the diagrams show which *raw* results fed into the calculation of the *category-* and *arousal-specific* results. Dotted lines and arrows indicate the results used for hypothesis testing. The hypothesis tests for response patterns shifting from the assigned to the experienced gender are presented on the top left (*category-specific* activation), top right (*raw* activation), and bottom left (*arousal-specific* activation) with their respective posterior probabilities. The central circle represents the support for the main hypothesis with the sectors fanning out representing the derived sub-hypotheses. The results for the sports scenes showing female (SF) or male (SM) athletes were only included in the hypothesis tests of the *arousal-specific* activation. The activation for heterosexual stimuli was contrasted to the average of SW and SM activations (S). AG: assigned gender, TX: transgender, EG: experienced gender, TW: trans women, TM: trans men, CW: cis women, CM: cis men, pre: pre-treatment assessment, post: post-treatment assessment, ⚤: scenes of male-female intercourse, ⚢: scenes of female-female intercourse, ⚣: scenes of male-male intercourse.


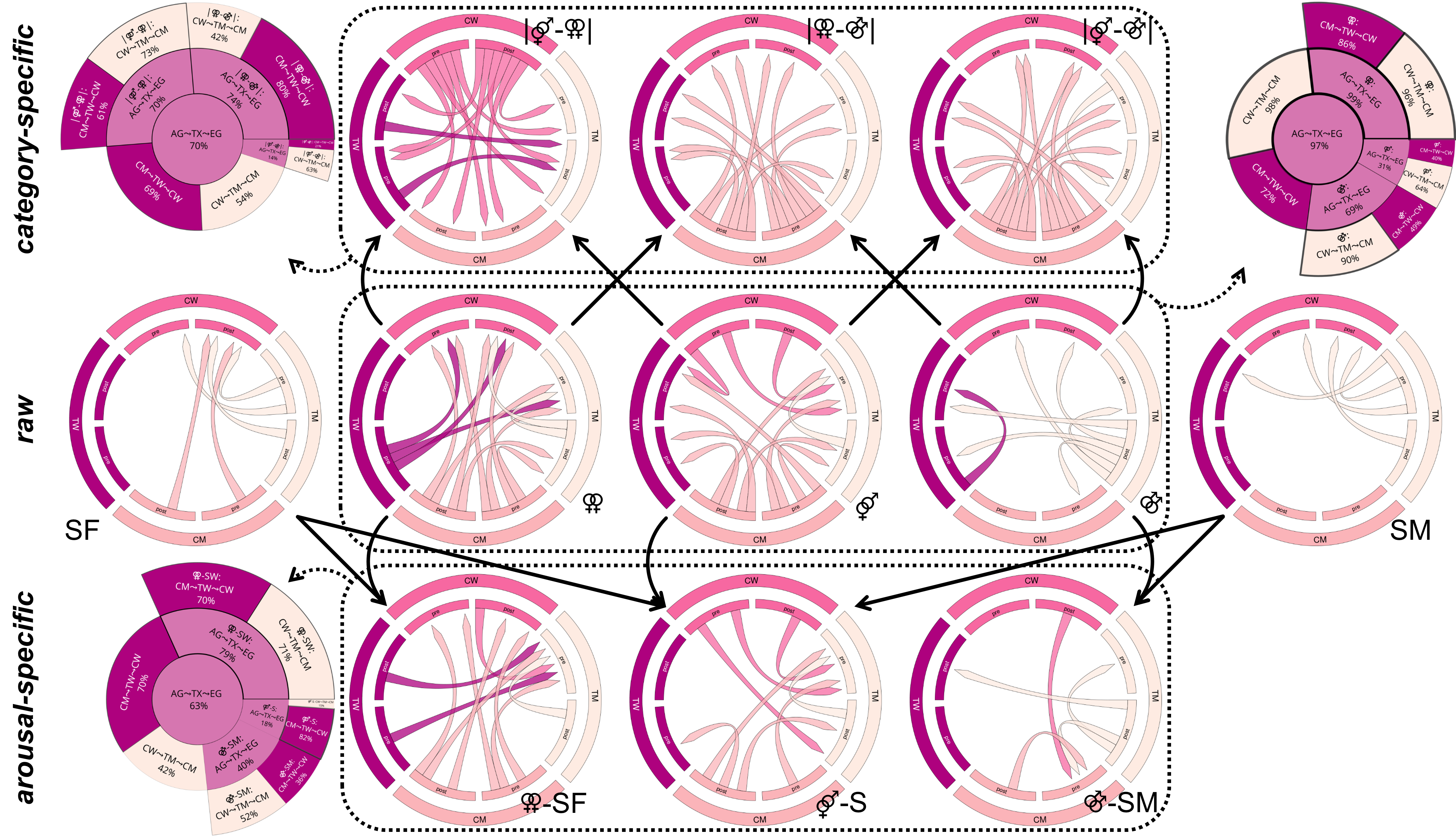


**Figure S3: Results of the behavioral analyses using flat priors including *arousal-specific* contrasts.** For visualization purposes, the chord diagrams (Gu et al., 2014) only show the differences between groups and time points with a 95% highest posterior density not covering 0. The top row shows the *category-specific*, the middle row the *raw*, and the bottom row the *arousal-specific* differences. Colored arrow directions in the chord diagrams indicate “greater than” relations (i.e., the arrows represent the “>” relation with the smaller value at the tip and the larger at the shaft). Black solid arrows between the diagrams show which *raw* results fed into the calculation of the *category-* and *arousal-specific* results. Dotted lines and arrows indicate the results used for hypothesis testing. The hypothesis tests for response patterns shifting from the assigned to the experienced gender are presented on the top left (*category-specific* responses), top right (*raw* responses), and bottom left (*arousal-specific* responses) with their respective posterior probabilities. The central circle represents the support for the main hypothesis with the sectors fanning out representing the derived sub-hypotheses. The results for the sports scenes showing female (SF) or male (SM) athletes were only included in the hypothesis tests of the *arousal-specific* responses. The responses to heterosexual stimuli were contrasted to the average of SW and SM responses (S). AG: assigned gender, TX: transgender, EG: experienced gender, TW: trans women, TM: trans men, CW: cis women, CM: cis men, pre: pre-treatment assessment, post: post-treatment assessment, ⚤: scenes of male-female intercourse, ⚢: scenes of female-female intercourse, ⚣: scenes of male-male intercourse.

# Supplementary discussion

After contrasting the subjective responses to the erotic stimuli with those to the sports stimuli, the overall support for a shift in sexual arousal patterns for the TW group stayed almost the same. However, at a closer look, this is the result of a general reduction in support for our hypothesis regarding female-female stimuli (now contrasted to sports scenes depicting two women) and an increase for the male-female stimuli, but only in TW. One explanation for the decrease in support for the female-female stimuli might be the different groups’ partially shared response pattern with the sports scenes depicting women. The higher decrease for TM (-26%) than TW (-14%), with the TM group showing marked differences to the CM and CW groups before GHT only for the female-female stimuli (see Figure S1, sports-female and female-female chord diagrams and the supplementary Excel files for detailed parameter estimates), can be seen as support for this assumption. This shared response pattern of the TW group for female-female and female sports stimuli, in turn, might stem from the subjects rating the sports scenes for general liking and not sexual arousal, which was necessary since the latter led to confusion in pilot measurements. Another, not mutually exclusive explanation for the decrease in support for our hypothesis is increased variance through subtraction of the responses leading to broader credible intervals (i.e., increased uncertainty) of the parameter estimates entering hypothesis testing. Shared response patterns between erotic and sports stimuli could also explain the reduction of support for the TM group.

For VS activation, where only the TW group originally showed strong support for a shift of the sexual arousal pattern in the hypothesized direction in the *raw* data, no noteworthy support remained for the *category-specific* or *arousal-specific* contrasts. The reason underlying this observation is likely again a shared response pattern, this time for VS activation in the TW group (see Figure S2, chord diagrams for the *raw* activation and the supplementary Excel files for detailed parameter estimates).

Even though sports scenes were argued as reasonable stimuli to control for various unspecific factors present when watching scenes of intercourse (e.g., general arousal, attention, observing interacting people; Ferretti et al. (2005)), many of the studies using this control focused on a single sex or gender (e.g., Bruce A. Arnow et al. (2002); B. A. Arnow et al. (2009); Brunetti et al. (2008); Safron et al. (2007, 2017)). A recent study in cis- and transgender individuals argued against the use of sports scenes as control because arousal (it seems that the authors are referring to sexual arousal in particular) cannot be ruled out (S. C. Mueller, Wierckx, & T'Sjoen, 2020). Also, in studies including CW and CM participants, either no control stimuli were used (Safron, Sylva, Klimaj, Rosenthal, & Bailey, 2020) or it was explicitly argued against them due to neuronal responses potentially depending on the sex of the athletes depicted (Sylva et al., 2013).

# Supplementary references

Akima, H. (1970). A New Method of Interpolation and Smooth Curve Fitting Based on Local Procedures. *J. ACM, 17*(4), 589–602. doi:10.1145/321607.321609

Arnow, B. A., Desmond, J. E., Banner, L. L., Glover, G. H., Solomon, A., Polan, M. L., . . . Atlas, S. W. (2002). Brain activation and sexual arousal in healthy, heterosexual males. *Brain, 125*(5), 1014-1023. doi:10.1093/brain/awf108

Arnow, B. A., Millheiser, L., Garrett, A., Lake Polan, M., Glover, G. H., Hill, K. R., . . . Desmond, J. E. (2009). Women with hypoactive sexual desire disorder compared to normal females: A functional magnetic resonance imaging study. *Neuroscience, 158*(2), 484-502. doi:https://doi.org/10.1016/j.neuroscience.2008.09.044

Beall, E. B., & Lowe, M. J. (2007). Isolating physiologic noise sources with independently determined spatial measures. *Neuroimage, 37*(4), 1286-1300. doi:10.1016/j.neuroimage.2007.07.004

Brunetti, M., Babiloni, C., Ferretti, A., Del Gratta, C., Merla, A., Olivetti Belardinelli, M., & Romani, G. L. (2008). Hypothalamus, sexual arousal and psychosexual identity in human males: a functional magnetic resonance imaging study. *Eur J Neurosci, 27*(11), 2922-2927. doi:10.1111/j.1460-9568.2008.06241.x

Ferretti, A., Caulo, M., Del Gratta, C., Di Matteo, R., Merla, A., Montorsi, F., . . . Romani, G. L. (2005). Dynamics of male sexual arousal: distinct components of brain activation revealed by fMRI. *Neuroimage, 26*(4), 1086-1096. doi:https://doi.org/10.1016/j.neuroimage.2005.03.025

Folch-Fortuny, A., Arteaga, F., & Ferrer, A. (2015). PCA model building with missing data: New proposals and a comparative study. *Chemometrics and Intelligent Laboratory Systems, 146*, 77-88. doi:https://doi.org/10.1016/j.chemolab.2015.05.006

Gizewski, E. R., Krause, E., Schlamann, M., Happich, F., Ladd, M. E., Forsting, M., & Senf, W. (2009). Specific cerebral activation due to visual erotic stimuli in male-to-female transsexuals compared with male and female controls: an fMRI study. *J Sex Med, 6*(2), 440-448. doi:10.1111/j.1743-6109.2008.00981.x

Gu, Z., Gu, L., Eils, R., Schlesner, M., & Brors, B. (2014). circlize Implements and enhances circular visualization in R. *Bioinformatics, 30*(19), 2811-2812. doi:10.1093/bioinformatics/btu393

Jørgensen, E., & Pedersen, A. R. (1997). How to obtain those nasty standard errors from transformed data - and why they should not be used. In. Biometry Research Unit, Department of Biometry and Informatics: Danish Institute of Agricultural Sciences.

Klöbl, M., Michenthaler, P., Godbersen, G. M., Robinson, S., Hahn, A., & Lanzenberger, R. (2020). Reinforcement and Punishment Shape the Learning Dynamics in fMRI Neurofeedback. *Front Hum Neurosci, 14*(304). doi:10.3389/fnhum.2020.00304

Ku, H. L., Lin, C. S., Chao, H. T., Tu, P. C., Li, C. T., Cheng, C. M., . . . Hsieh, J. C. (2013). Brain signature characterizing the body-brain-mind axis of transsexuals. *PLoS One, 8*(7), e70808. doi:10.1371/journal.pone.0070808

Mueller, K., Lepsien, J., Möller, H. E., & Lohmann, G. (2017). Commentary: Cluster failure: Why fMRI inferences for spatial extent have inflated false-positive rates. *Front Hum Neurosci, 11*, 345. doi:10.3389/fnhum.2017.00345

Mueller, S. C., Wierckx, K., & T'Sjoen, G. (2020). Neural and Hormonal Correlates of Sexual Arousal in Transgender Persons. *J Sex Med, 17*(12), 2495-2507. doi:https://doi.org/10.1016/j.jsxm.2020.08.021

Patel, A. X., Kundu, P., Rubinov, M., Jones, P. S., Vértes, P. E., Ersche, K. D., . . . Bullmore, E. T. (2014). A wavelet method for modeling and despiking motion artifacts from resting-state fMRI time series. *Neuroimage, 95*(100), 287-304. doi:10.1016/j.neuroimage.2014.03.012

Safron, A., Barch, B., Bailey, J. M., Gitelman, D. R., Parrish, T. B., & Reber, P. J. (2007). Neural correlates of sexual arousal in homosexual and heterosexual men. *Behav Neurosci, 121*(2), 237-248. doi:10.1037/0735-7044.121.2.237

Safron, A., Sylva, D., Klimaj, V., Rosenthal, A. M., & Bailey, J. M. (2020). Neural Responses to Sexual Stimuli in Heterosexual and Homosexual Men and Women: Men's Responses Are More Specific. *Arch Sex Behav, 49*(2), 433-445. doi:10.1007/s10508-019-01521-z

Safron, A., Sylva, D., Klimaj, V., Rosenthal, A. M., Li, M., Walter, M., & Bailey, J. M. (2017). Neural Correlates of Sexual Orientation in Heterosexual, Bisexual, and Homosexual Men. *Sci Rep, 7*, 41314. doi:10.1038/srep41314

Sylva, D., Safron, A., Rosenthal, A. M., Reber, P. J., Parrish, T. B., & Bailey, J. M. (2013). Neural correlates of sexual arousal in heterosexual and homosexual women and men. *Horm Behav, 64*(4), 673-684. doi:10.1016/j.yhbeh.2013.08.003
